# Chromatin structure and transposable elements in organismal aging

**DOI:** 10.3389/fgene.2013.00274

**Published:** 2013-12-04

**Authors:** Jason G. Wood, Stephen L. Helfand

**Affiliations:** Department of Molecular Biology, Cell Biology, and Biochemistry, Brown UniversityProvidence, RI, USA

**Keywords:** chromatin, heterochromatin, transposable elements, retrotransposons, RNAi, histone modifications, epigenetics of aging, silencing

## Abstract

Epigenetic regulatory mechanisms are increasingly appreciated as central to a diverse array of biological processes, including aging. An association between heterochromatic silencing and longevity has long been recognized in yeast, and in more recent years evidence has accumulated of age-related chromatin changes in *Caenorhabditis elegans, Drosophila*, and mouse model systems, as well as in the tissue culture-based replicative senescence model of cell aging. In addition, a number of studies have linked expression of transposable elements (TEs), as well as changes in the RNAi pathways that cells use to combat TEs, to the aging process. This review summarizes the recent evidence linking chromatin structure and function to aging, with a particular focus on the relationship of heterochromatin structure to organismal aging.

## INTRODUCTION

In addition to the genetic information encoded in the nuclear DNA genome, cells are also able to store information in the form of epigenetic modifications to proteins and nucleic acids that affect cellular processes and phenotypes. Some of these epigenetic mechanisms include methylation, acetylation, and other covalent modifications of histones and other proteins, methylation of the DNA itself, and posttranscriptional regulation of both coding and non-coding RNA species. In recent years, epigenetic regulation has begun to be recognized as playing a key role in a host of biological processes, including aging. Unlike genetic changes such as base pair deletion or mutation, epigenetic changes are generally reversible and thus represent not only an easy mechanism for cells to regulate various processes, but also an attractive therapeutic target for potential intervention in human disease states.

Many of the most prominent epigenetic modifications, including covalent post-translational modification of histones and chromatin remodeling, take place in the context of chromatin. The basic structure of chromatin consists of DNA wrapped around a histone octamer core, forming a nucleosome. The repeating nucleosome units are then packaged together and condensed in a complex higher order structure. The highly conserved core histone proteins (histones H2A, H2B, H3, and H4) contain N-terminal tails that are rich in lysine residues, which are prime targets for a number of covalent posttranslational modifications, including acetylation, methylation, and phosphorylation. Modification of these residues creates a “histone code” that serves a number of important regulatory functions, including recruitment of transcription factors and polymerases, and assembly of chromatin complexes that either promote or repress transcription. Histone marks are added and removed by a host of methyl- and acetyltransferases, and deacetylases and demethylases, which frequently have catalytic activity specific to a single residue. Chromatin structure is not uniform throughout the genome, and is usually classified into two types: euchromatin and heterochromatin. Euchromatin is generally transcriptionally active, gene rich, accessible to polymerases and transcription factors, and enriched for “active” histone marks such as H3K4 and H3K36 methylation and H4K16 acetylation. Heterochromatin domains, on the other hand, are normally gene poor, transcriptionally repressed, tightly packed and inaccessible to transcription factors, and enriched for “repressive” histone marks such as H3K9 or H3K27 methylation.

In this review we highlight evidence from the recent literature linking changes in chromatin structure and function to the aging process. We discuss early theories of chromatin change with age, highlighting pioneering work in the yeast *S. cerevisiae*. We also discuss the role of chromatin in the replicative senescence model of cellular aging. We then examine evidence for chromatin changes in aging in animal model systems, including *Caenorhabditis elegans*, *D. melanogaster*, mice, and rats. We discuss the important regulatory role of the spatial organization of chromatin within the nucleus, and how it may contribute to aging both at the organismal and cellular level. Finally, we review the link between chromatin and transposable elements, and discuss how changes in expression and activity of transposable elements may be relevant to the aging process.

## HETEROCHROMATIN LOSS MODEL OF AGING

It has long been supposed that epigenetic effects influence the aging process (Macieira-Coelho, 1991; Kennedy et al., 1995). To date there have been a number of studies looking at how the structure of chromatin changes as organisms age (Oberdoerffer and Sinclair, 2007). Much of the early work linking chromatin to aging was done in the budding yeast *S. cerevisiae*. Because yeast is a single-celled organism, one of the ways aging is measured in this organism is to assay replicative lifespan, or the number of daughter cells each new mother cell can produce by budding before entering a non-dividing senescent state. In experiments performed in the 1990s, it was observed that aging and consequent sterility in yeast was correlated with a loss of characteristic heterochromatic silencing at telomeres, mating type locus, and ribosomal DNA (rDNA) repeats (Kim et al., 1996; Smeal et al., 1996; Kennedy et al., 1997). The association between chromatin and aging was strengthened when Sir2, which had originally been identified in a screen for factors involved in heterochromatic genetic silencing, was found to be associated with a longevity phenotype in budding yeast (Kaeberlein et al., 1999). Sir2 was subsequently characterized enzymatically as a NAD^+^-dependent histone deacetylase (Frye, 2000; Imai et al., 2000; Landry et al., 2000), and later the sirtuin family (Sir2 homologues) was found to have a wide range of activities and targets (reviewed in Haigis and Sinclair, 2010). This early yeast work lent support to the heterochromatin loss model of aging, where heterochromatin structure within the genome breaks down or weakens as an organism or cell ages (Villeponteau, 1997; Tsurumi et al., 2012). The loss of heterochromatin is expected to have deleterious effects on cellular homeostasis, for instance by causing aberrant expression of normally repressed genes, or redirection of limited energy toward repair or maintenance of damaged heterochromatin regions. Inappropriate transcription of silenced genes may cause cellular damage through a number of different mechanisms. An increase in transcriptional noise may cause cumulative harm by appropriating cellular factors necessary for transcribing and expressing normal genes. Alternatively, loss of silencing may lead to expression of certain genes that are causative of aging phenotypes, such as in yeast when silent mating cassette derepression leads to co-expression of both mating type genes, causing sterility and senescence (Smeal et al., 1996). However, the specific consequences of loss of heterochromatin silencing and the potential role of the transcriptional and translational machinery in downstream effects remain poorly characterized.

## CHROMATIN STRUCTURE IN YEAST AGING

In more recent years, a number of yeast studies have looked at changes in histone structure and histone post-translational modifications with age (reviewed in Feser and Tyler, 2011). In yeast cells, levels of H4K16 acetylation increase with age, and this is accompanied by a decrease in Sir2 levels (Sir2 deacetylates H4K16ac; Dang et al., 2009). In this study, aging was also associated with a loss of histones in certain heterochromatic regions, and a concomitant decrease in silencing at these loci. In addition, a study by Feser et al. found that yeast cells lacking the histone chaperone Asf1 had a shortened lifespan, and this was due to a defect in histone H3K56 acetylation and a failure to properly regulate histone levels (Feser et al., 2010). This study also reported that histone protein levels themselves decline with age. Interestingly, when histone levels were raised artificially, either by knocking out *HIR1*, which represses histone transcription, or by overexpressing histones H3 and H4 directly, lifespan was increased. Together with earlier work, these studies support a strong link between aging and chromatin structure in yeast, and suggest that aging results at least in part from a failure to maintain proper chromatin structure with time.

## CHROMATIN IN REPLICATIVE SENESCENCE

The link between chromatin structure and aging has also been examined in the replicative senescence of tissue culture cells. Cells in culture will divide a finite number of times before ceasing mitosis and entering into a non-dividing quiescent state, a process termed replicative senescence. In a way, experiments in this system are akin to the measurement of replicative lifespan commonly performed in budding yeast. A number of age-related changes in chromatin structure have been observed in senescent cells (O’Sullivan and Karlseder, 2012). Histone levels are known to decline during replicative senescence, and several histone methylation and acetylation marks redistribute in senescent cells (O’Sullivan et al., 2010; Ivanov et al., 2013). As cells senesce, they also form regions of dense non-pericentromeric heterochromatin termed senescence-associated heterochromatin foci (SAHF), which are characterized by a high level of repressive heterochromatin histone marks such as H3K9me3 and H3K27me3 (Narita et al., 2003; Zhang et al., 2005; Kosar et al., 2011; Chandra et al., 2012). A recent study by Shah et al. examined large scale chromatin changes in senescent cells with a ChIP-seq approach (Shah et al., 2013). When compared with proliferating cells, in senescent cells both H3K4me3 and H3K27me3 were enriched in large contiguous regions that correlated with lamin B1-associated domains (LADs). Areas of contiguous negative enrichment of H3K27me3 were also observed and tended to be rich in genes and promoters. Interestingly, senescence associated changes in these histone marks were also correlated with senescence associated gene expression changes, with loss of H3K4me3 at down-regulated genes, and loss of H3K27me3 at up-regulated genes (Shah et al., 2013).

De Cecco et al. (2013) examined replicatively senescent cells using the FAIRE (formaldehyde-assisted isolation of regulatory elements) technique (Giresi and Lieb, 2009), which measures open (transcriptionally active) and closed (transcriptionally silent) chromatin states. The technique exploits the differential partitioning during phenol/chloroform extraction of DNA fragments containing densely packed histones (more protein, more likely to be in organic phase) or relatively sparse histones (more DNA, more likely to be in aqueous phase). They observed that replicatively senescent cells tend to show a “smoothing” of chromatin relative to dividing cells (De Cecco et al., 2013). In other words, regions of open and closed chromatin are less distinct and look more similar to each other in senescent cells. Promoters and enhancers of genes that are active in dividing cells become more closed upon senescence, and regions of normally silent heterochromatin become more open. Results from these replicative senescence studies suggest that structural changes in chromatin with age are an important conserved characteristic in metazoans.

## AGE AND CHROMATIN IN ANIMAL MODEL SYSTEMS

### C. elegans

In addition to evidence accumulating from yeast and cellular senescence models of aging, several studies in animal model systems have assessed changes in chromatin structure during organismal aging. In the roundworm *C. elegans*, a number of recent studies have demonstrated a relationship between lifespan and certain histone modifications. Disruption of the ASH-2 complex in worms, which contains a H3K4 methyltransferase, led to an increase in worm lifespan (Greer et al., 2010). Conversely, disruption of RBR-2, a H3K4 demethylase, led to a reduced lifespan and supported the conclusion that excessive H3K4 trimethylation, a hallmark of actively transcribed chromatin, was detrimental to lifespan. A follow up study showed that the effects of extended lifespan induced by these genetic modifications were heritable up to the third generation (Greer et al., 2011).

In addition to changes in active chromatin marks such as H3K4me3, there have also been reports of associations between aging and heterochromatin marks. Knockdown of the worm ortholog of LSD1, a histone demethylase that targets methylated H3K4 and H3K9 residues, led to a significant increase in lifespan (McColl et al., 2008). The H3K27me3 mark is found in association with regions of Polycomb heterochromatin, a type of repressive heterochromatin responsible for regulating silencing of numerous genes during development. A pair of studies examined the role of H3K27 methylation in worm aging, and found that disruption of the H3K27me3 demethylase UTX-1 led to increased levels of H3K27me3 marks in the genome as well as increased lifespan (Jin et al., 2011; Maures et al., 2011). Normal aging also showed an increase in UTX-1 activity, as well as a decline in H3K27me3 methylation. The lifespan extension was dependent on a functional *daf-16* gene, suggesting the effect functions through the insulin signaling pathway. Together, these *C. elegans* studies showed that aging is associated with a number of histone changes, and that genetic intervention to directly modulate histone marks can lead to changes in lifespan.

### Drosophila melanogaster

The fruit fly *Drosophila melanogaster* has also been used as a model system to study chromatin and aging. A whole genome study of aging fly heads showed several chromatin changes that occurred with age (Wood et al., 2010). Marks typifying active chromatin, such as H3K4me3 and H3K36me3, showed a general decline across genes with age. However, the most notable change was a significant decrease with age in the enrichment of the repressive heterochromatin mark H3K9me3 as well as HP1 (heterochromatin protein 1, a component of heterochromatin) at pericentric heterochromatin loci. Changes in nuclear localization and organization of both H3K9me3 and HP1 were also observed. Additionally, genes that lost H3K9me3 or HP1 with age trended toward increased expression. In another recent study, Larson et al. genetically manipulated expression of HP1, and observed that flies with decreased HP1 expression exhibited shortened lifespan (Larson et al., 2012). Conversely, flies overexpressing a transgenic HP1 showed an increase in lifespan (Larson et al., 2012), although an earlier study using a free duplication containing the HP1 locus failed to observe lifespan extension (Frankel and Rogina, 2005). Flies with decreased HP1 also showed premature muscle degeneration compared with controls, while HP1 overexpressing flies showed increased muscle function and structure with age (Larson et al., 2012). In addition, old flies showed an overall decrease in heterochromatin as well as nuclear reorganization of HP1. Flies with decreased HP1 expression also showed a dramatic increase in ribosomal RNA (rRNA) transcripts, while flies overexpressing HP1 had a decline in such transcripts, demonstrating that disruption of heterochromatinstructure is sufficient to modulate transcription from the repetitive rDNA locus. These *Drosophila* data fit well with the heterochromatin loss model of aging described above, whereby loss of repressive heterochromatin leads to loss of silencing and aberrant gene expression, with consequent deleterious effects on the cell or organism. However, not all reported studies are consistent with this model. In contrast to the worm H3K27me3 studies described above (Jin et al., 2011; Maures et al., 2011), a study examining genetic mutations in the H3K27 histone methyltransferase component of the Polycomb complex in *Drosophila* reported that loss of function of this gene led to a decrease in H3K27 methylation, as well as an increase in lifespan (Siebold et al., 2010). These sometimes conflicting results underscore the possibility that there may be important tissue specific differences or differences between species in how chromatin marks are processed and their consequent effects on aging.

### MAMMALS

Age-related chromatin changes have also been observed in mammalian model systems, primarily mouse and rat. Most mammalian studies have tended to focus on changes in histone acetylation with age. A recent study of chromatin structure with age in the mouse cochlea showed a decrease in histone acetylation and an increase in histone methylation in aged mice compared to young mice (Watanabe and Bloch, 2013). Notable differences in DNA methylation patterns have also been observed between young and old mice, and these methylation changes are consistent across a number of different tissue types (Maegawa et al., 2010). In addition to direct observation of epigenetic marks in aging tissue, several studies have identified a role for histone modifications in functional and behavioral studies of mouse aging. Using a neurodegeneration mouse model allowing for targeted induction of neuronal loss, Fischer et al. examined the molecular basis for the environmental enrichment (EE) paradigm that is known to increase synaptic function and memory formation (Fischer et al., 2007). In this study, EE led to a significant increase in learning and memory among mice that had undergone neuronal loss, and this was correlated with an increase in histone acetylation and methylation at a number of different residues on histones H3 and H4. Furthermore, treatment with sodium butyrate, a histone deacetylase inhibitor, was sufficient to increase learning and memory in these mice (Fischer et al., 2007). In a similar study, this time examining age-related cognitive decline rather than induced neurodegeneration, it was observed that the brains of aged mice when compared with younger mice exhibited a hypoacetylation of H4K12 in the hippocampus, with a concomitant failure to activate a gene expression program necessary for memory consolidation (Peleg et al., 2010). When aged mice were treated with a histone deacetylase inhibitor in the hippocampus, H4K12 acetylation patterns, memory associated gene expression profiles, and learning behavior were all restored. Similarly, a study of aging rat brains showed reduced histone acetylation of H3K9 and H4K12 in the hippocampus with age, resulting in compromised expression of genes important for long-term potentiation (LTP; Zeng et al., 2011). Again, when rats were treated with histone deacetylase inhibitors, both acetylation levels as well as age-related LTP declines showed improvement.

Chromatin structure has also been implicated in mouse models of age-associated disease. In the APP/PS1 mouse model of Alzheimer’s disease, a decrease in histone H4 acetylation as well as defects in LTP and memory formation was observed when compared to control mice (Francis et al., 2009). Interestingly, treatment with histone deacetylase inhibitors restored acetylation levels as well as cognitive function in the affected mice. A second study in this model confirmed that treatment with histone deacetylase inhibitors was able to correct age-related memory impairment (Kilgore et al., 2010). In a mouse model of Hutchinson–Gilford progeria, which shows defective lamin A processing and an associated short lifespan, H4K16 is hypoacetylated, and this is also observed during normal aging (Krishnan et al., 2011). Furthermore, either overexpression of the histone acetyltransferase Mof or chemical inhibition of histone deacetylase activity caused amelioration of age-related symptoms and extension of lifespan in this short-lived model.

While most of the mammalian model system data has tended to show a decrease in histone acetylation with age, as noted above Feser et al. observed an increase with age of the H4K16ac mark in yeast (Feser et al., 2010). In yeast, the H4K16ac mark is particularly associated with the mating type loci and subtelomeric regions that are normally silenced by the SIR complex, where Sir2 acts to deacetylate H4K16 (Millar and Grunstein, 2006). It is likely that the observed increase in H4K16 acetylation is indicative of the loss of silencing in these regions that is known to take place with age (Kennedy et al., 1997). In multicellular organisms, the situation is likely significantly more complex, and there may also be cell type or tissue specific effects that lead to the apparently contradictory results. Importantly, the identification of age-related chromatin changes in not only replicative senescence models such as yeast and tissue culture, but also in animal models of organismal aging, argues strongly for chromatin structure playing an important conserved role in regulating the aging process.

## SPATIAL ORGANIZATION OF CHROMATIN

Chromatin is organized into discrete domains of euchromatin and heterochromatin within the linear genome. However, the spatial organization of chromatin within the nucleus also has important ramifications for regulation of gene expression (for recent reviews see Oberdoerffer and Sinclair, 2007; Bickmore and van Steensel, 2013; Gibcus and Dekker, 2013). It has long been appreciated that euchromatin and heterochromatin are not distributed equally throughout the volume of the nucleus. In yeast, heterochromatic telomere regions are preferentially associated with the nuclear envelope (Gotta et al., 1996). Furthermore, silencing of yeast chromatin can be switched on and off by targeting it to or away from the nuclear periphery (Andrulis et al., 1998; Feuerbach et al., 2002). In higher eukaryotes, chromatin regions termed lamin-associated domains (LADs) are similarly localized to the nuclear periphery, where they interact with the nuclear lamina. These LADs tend to be heterochromatic, gene poor, and refractory to transcription, and have been characterized in both mammalian as well as *Drosophila* cells (Pickersgill et al., 2006; Guelen et al., 2008). Interestingly, movement of these LADs away from the nuclear periphery is correlated with altered gene expression patterns (Peric-Hupkes et al., 2010). In addition to heterochromatin organization, transcriptionally active euchromatic regions are also located in distinct spatial domains within the nucleus known as transcription factories (Fraser and Bickmore, 2007; Rieder et al., 2012; Gibcus and Dekker, 2013).

Changes in nuclear architecture and spatial organization of chromatin have also been linked to both cellular senescence and organismal aging. Hutchinson-Gilford Progeria Syndrome (HGPS) is a rare genetic disorder characterized by symptoms that resemble premature aging, and is caused by mutations in the LMNA gene, which encodes lamin A, a key component of the nuclear lamina. These mutations lead to alternative splicing and incorrect processing of lamin A, resulting in a dominant negative form of lamin A called progerin that disrupts proper nuclear architecture, leading to misshapen nuclei, changes in heterochromatin organization, and defects in DNA replication and transcription (Eriksson et al., 2003; Goldman et al., 2004). Phenotypes similar to those observed in HGPS have also been observed in normal aging in fibroblasts taken from older individuals. These age-related changes include altered lamin A processing and nuclear structure (Scaffidi and Misteli, 2006; McClintock et al., 2007). In addition, expression of the nuclear lamin B1 decreases naturally as cells enter senescence (Shimi et al., 2011; Freund et al., 2012; Shah et al., 2013). Furthermore, in their recent study on lamin B1 and chromatin, Shah et al. observed that knocking down lamin B1 expression causes both entry into senescence as well as large scale reorganization of LAD chromatin structure, including regions of H3K4me3 and H3K27me3 marks (Shah et al., 2013).

Age-related changes in nuclear structure have also been described in animal models. In *C. elegans*, nuclear structure undergoes several changes with age, including loss of peripheral heterochromatin and deterioration of nuclear shape and structure. Genetically reducing levels of lamin in the worm also leads to a shortened lifespan (Haithcock et al., 2005). Together these data suggest that breakdown of nuclear architecture is a hallmark of the aging process. However, the details of exactly how spatial organization of chromatin within the nucleus affects regulation of gene expression remain enigmatic. Techniques based on chromosomal conformation capture (3C), including the derivatives 4C, 5C, and Hi-C, have been informative in identifying distal chromatin interactions in a number of organisms. In this family of techniques, chromatin is crosslinked together with formaldehyde, fragmented, and ligated, then examined by a number of methods (most recently high throughput sequencing) for novel ligation products that indicate long range interactions between spatially distinct chromatin domains, representing potentially important regulatory associations (Gibcus and Dekker, 2013). A spatial mapping of the entire human genome using the Hi-C technique at 1 mb resolution revealed a segregation of the nucleus into two compartments corresponding to open and closed chromatin (Lieberman-Aiden et al., 2009). The observed chromatin architecture was consistent with a “fractal globule” model where stretches of open or closed chromatin are packed together in dense clusters and organized into larger compartments, with greater interaction within each compartment than across compartments. The *S. cerevisiae* genome was mapped in a similar manner with 4C, revealing a three dimensional structure where the centromeres clustered together at one end of the nucleus, telomeres exhibited both intra- and interchromosomal associations, and rDNA repeats were organized into a distal nucleolus (Duan et al., 2010). Furthermore, interchromosomal interactions were common and include tRNA genes, replication origins, and known sites of chromosomal breakage. Continued use of high resolution techniques such as these will be important for unraveling the complex three dimensional interactions of chromatin, and determining how changes in higher order chromatin organization contribute to aging and age-related phenotypes.

## CHROMATIN AND TRANSPOSABLE ELEMENTS

Heterochromatin establishment and maintenance is important for proper silencing to prevent inappropriate gene expression. One common class of transcripts that could have potentially deleterious effects when expressed is transposable elements (TEs). Most eukaryotic genomes contain a large amount of non-coding DNA that is derived from TEs (reviewed in Slotkin and Martienssen, 2007). For a long time most of these sequences were dismissed as “junk DNA,” although in recent years it has become clear that TEs are much more biologically interesting than originally thought, and are in fact widely transcriptionally expressed and can regulate nearby genes (Faulkner et al., 2009). TEs are sequences of DNA that can move within the genome, and are generally divided into two classes: Class I (retrotransposons) and Class II (DNA transposons). The retrotransposon class includes two subgroups: the LTR (long terminal repeat) type, which is flanked by direct repeats, and the non-LTR type, which includes LINEs (long interspersed elements) and SINEs (short interspersed elements). Retrotransposons propagate in a manner similar to retroviruses: they are transcribed by the host cell machinery to an RNA intermediate, encode their own reverse transcriptase which makes a DNA copy of the element, and then are integrated at a new location in the genome. This represents a “copy and paste” mechanism – the old transposon remains in place, and successful retrotransposition results in a new copy and an expanded host genome. DNA transposons, on the other hand, are flanked by inverted repeats, and are excised from the DNA and integrated at a new location by a transposase, which they frequently self encode. DNA transposons thus transpose with a “cut and paste” mechanism that is generally not duplicative, although excision is frequently imperfect and can leave behind partial sequences in the genome. TEs or remnants of TEs make up a large proportion of eukaryotic genomes, generally from 30 to 80% depending on species (detailed in Kidwell, 2002). The human genome, for example, contains about 50% TE material, and in some plants the vast majority of the genome consists of TEs (Kidwell and Lisch, 1997; Slotkin and Martienssen, 2007). The relative proportion of different types of TEs also varies appreciably between species. For example, the *Drosophila* genome has a large number of LTR type retrotransposons, whereas the human genome is characterized by abundant LINEs and SINEs, such as the LINE1 element that makes up 17% of the genome (Slotkin and Martienssen, 2007).

Most TEs, especially those that have been present in the genome for a very long time, are not active and have accumulated mutations that make them incapable of active transposition (Faulkner et al., 2009). However, even inactive TEs are still capable of initiating transcription from promoters contained within the element (Conley et al., 2008). TEs are known to be expressed in response to a number of organismal and cellular stresses, including DNA damage, UV, radiation, temperature, wounding, and infection (reviewed in Capy et al., 2000). In addition to DNA damage from radiation, increased concentrations of reactive oxygen species have also been shown to induce expression of TEs (Bradshaw and McEntee, 1989; Ikeda et al., 2001; Stoycheva et al., 2010). A recent study in rats used an acute stress paradigm to examine H3K9 methylation and TE expression in the hippocampus (Hunter et al., 2012). In response to stress, H3K9me3 levels increased throughout the hippocampus, but particularly at TE loci, and this was correlated with a repression of expression of certain TEs. TEs are also known to have significant effects on endogenous gene expression, both transcriptionally and post-transcriptionally, through a number of different mechanisms including sense or antisense transcriptional activation, induction of heterochromatic silencing, small RNA targeting, and alternative splicing (reviewed in Feschotte, 2008).

Active, mobile TEs can be highly mutagenic and cause genomic instability, and consequently in order to combat these effects cells have evolved strategies to suppress TE activity. The cellular RNAi machinery prevents TE mobilization by multiple mechanisms (reviewed in Castel and Martienssen, 2013). Details vary by organism and source of RNA, but in general dsRNAs targeted for silencing are processed by Dicer enzymes into short endogenous small interfering RNAs (esiRNAs), and subsequently loaded into an Argonaute (Ago)-containing complex known as RISC (RNA-induced Silencing Complex; reviewed in Pratt and MacRae, 2009). RISC complexes can induce silencing in at least two ways. First, these complexes are known to bind chromatin and repress transcription of their targets by catalyzing formation and spreading of silencing heterochromatin (Verdel et al., 2004; Bühler et al., 2006). This is accomplished by recruiting heterochromatin proteins and histone methyltransferases, resulting in H3K9 methylation that is characteristic of transcriptionally silent heterochromatin (Bühler et al., 2006). This mechanism has been well described in yeast, but appears to be widely conserved, including in mammalian cells (reviewed in Sampey et al., 2012). In mammals the siRNA pathway induces transcriptional gene silencing via DNA methylation as well as H3K9 methylation, and this silencing requires both Ago1 as well as active RNA polymerase II (Morris et al., 2004; Kim et al., 2006; Weinberg et al., 2006). Secondly, in addition to transcriptional gene silencing, RISC complexes perform post-transcriptional silencing of target RNAs by recognizing and hydrolyzing target transcripts, again in a widely conserved mechanism. A catalytically active Ago protein (e.g., Ago2 in humans) binds “guide” siRNAs and “target” RNA transcripts, and subsequently degrades the target with a slicer RNase catalytic activity (Liu et al., 2004; Meister et al., 2004; Gregory et al., 2005; Matranga et al., 2005; Rand et al., 2005). Given the potential mutagenic effects of TE expression and mobilization, these RNAi silencing mechanisms offer an important cellular defense mechanism against genomic damage.

## TRANSPOSABLE ELEMENTS IN AGING

**FIGURE 1:**
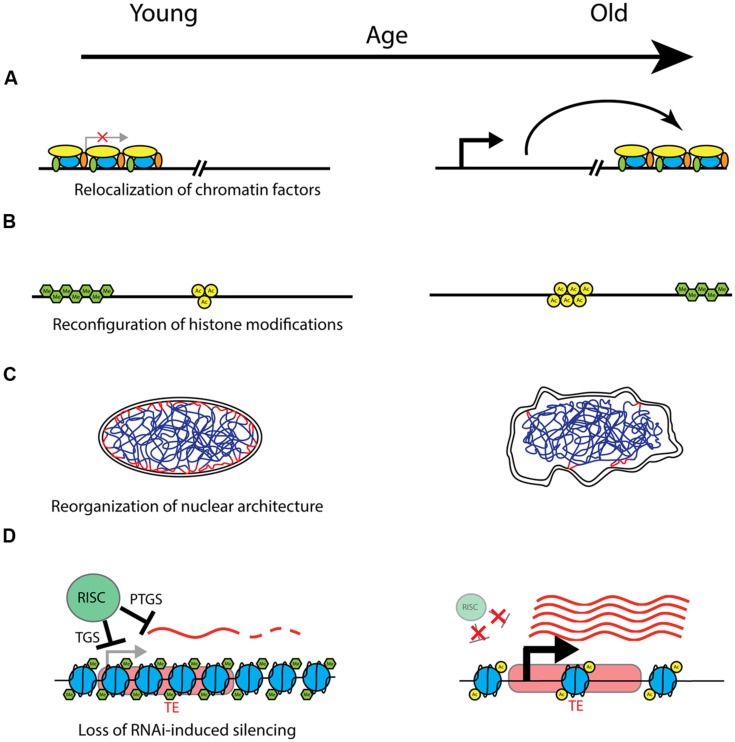
**Models of potential chromatin-related mechanisms of aging.** Changes that may take place with age are shown with young on the left, and old on the right. **(A)** With age, chromatin factors such as silencing complexes may be relocalized to different areas of the genome, leading to location-dependent changes in silencing and gene expression. **(B)** Chromatin domains characterized by particular posttranslational histone modifications (here represented generically with green hexagons for methylation, and yellow circles for acetylation) may change in both location and extent with age, with corresponding effects on expression of underlying genes. Possible mechanisms include spreading, shrinking, removal, or establishment of repressive heterochromatin domains by methyltransferases and demethylases (e.g. H3K9me3 domains), and changes in chromatin accessibility due to chromatin remodeling factors. **(C)** The structure and integrity of the nucleus declines with age, and this may have important effects on the spatial organization of chromatin, with consequent effects on silencing and gene expression. Depicted is a nucleus with interphase chromatin. Heterochromatic LADs (lamin-associated domains) are marked in red, where they associate with the nuclear lamina on the inner nuclear membrane. Degradation of nuclear architecture with age could lead to failure to maintain silent heterochromatin at LADs with age. **(D)** Model of transposable element (TE) derepression with age. In young healthy cells, transcription and expression from TEs (represented here in red) is prevented by the RNAi machinery. The RNAi-induced silencing complex (RISC) performs transcriptional gene silencing (TGS) by recruiting chromatin remodeling factors and establishing refractory heterochromatin, shown here as densely packed nucleosomes with characteristic histone modifications (e.g., H3K9me3). Furthermore, any transcripts that are produced (represented by wavy red lines) are targeted for degradation by a posttranscriptional gene silencing (PTGS) mechanism also mediated by the RISC. With age, declines in the functionality or efficiency of small RNA processing and targeting may lead to a breakdown in these silencing mechanisms. The chromatin structure is modified, with characteristics of more actively expressed chromatin, such as fewer histone methylation (H3K9me) marks, and increased histone acetylation marks and chromatin accessibility. Additionally, loss of PTGS can lead to increased levels of TE transcripts, which can undergo transposition and land in other areas of the genome, with deleterious effects.

Because TEs are particularly enriched in heterochromatin, and heterochromatin changes have been linked to the aging process, one attractive hypothesis is that aging may be due in part to increasing inability to control and prevent TE expression and mobilization (Driver and McKechnie, 1992). In fact, because heterochromatin is gene poor, but rich in TEs, TE expression may be the major consequence of the age-related breakdown in heterochromatin structure. A schematic of how this may happen is presented in **Figure 1D**. In support of this idea, St. Laurent et al. argue in a recent review that transposition of LINE/L1 elements, which are particularly prevalent in mammals and are activated by stress, may play an important role in mammalian aging and evolution (St Laurent et al., 2010). In this model, LINE activation leads not only to mutagenic insertions, but also other DNA damage that accumulates with age and can cause genomic instability even in the absence of a successful transposition event. Furthermore, they propose an antagonistic pleiotropy explanation for retrotransposon mobilization, whereby LINE activation provides an evolutionary advantage by allowing for rapid stress-induced genetic variation, but can also lead to reduced individual lifespan as cellular damage accumulates.

Several recent studies support the involvement of TEs in the aging process. Using a chronological aging model, a study in budding yeast observed that the yeast retrotransposon Ty1 showed increased mobility with age, and this was correlated with chromosome rearrangements and other hallmarks of genomic instability (Maxwell et al., 2011). Furthermore, interventions that decreased transposon mobility also exhibited a decrease in age-related deleterious genomic instability. In *C. elegans*, expression of the endogenous retrotransposon *Cer1* (gypsy/Ty3 family) has been shown to increase with age (Dennis et al., 2012). Capsid particles from *Cer1* accumulate on microtubules in meiotic germ cells of older animals, and the capsids likely use these microtubules to target host cell nuclei. Retrotransposons are also active in the mammalian nervous system during neural development, and influence neural cell fate by altering expression of neural genes (Muotri et al., 2005). In addition, retrotransposons are also active in several regions of the adult brain, leading to somatic mosaicism (Coufal et al., 2009; Baillie et al., 2011). A recent study examining epigenetic changes in the aging mouse brain observed differences in DNA methylation as well as some histone modifications at repetitive DNA elements with age (Ryu et al., 2011). Specifically, overall histone acetylation of both H3 and H4 decreased with age at several TEs as assayed by ChIP-qPCR, and some histone methylation marks, such as H3K9me3 and H4K20me3, were also decreased in some TEs.

Transposable elements have also been implicated in cellular senescence. As human adult stem cells undergo senescence, DNA damage becomes concentrated in hot spots within TEs, and there is a noted increase in transcription of *Alu* elements (Wang et al., 2011; De Cecco et al., 2013). When *Alu* transcripts were targeted for degradation in these cells by shRNA, the cells were able to reverse senescent phenotypes and begin proliferating, demonstrating that TE expression can cause cellular toxicity (Wang et al., 2011). De Cecco et al. (2013) observed a number of TEs that were enriched in late stage senescent cells when examined with FAIRE-seq (which measures chromatin accessibility), including not only *Alu* elements but also L1, SVA, and satellite sequences. Interestingly, the evolutionarily more recent L1 elements, which are more likely to be capable of retrotransposition, showed the highest enrichment in late senescent cells. In accordance with the observed increase in FAIRE-seq signal of L1 elements, both RNA levels as well as genomic copy number increased in senescent cells, suggesting mobilization and transposition of these elements during senescence. Additionally, a pair of recent studies examined the role of *Alu* elements and Dicer1 function in age-related macular degeneration (Kaneko et al., 2011; Tarallo et al., 2012). Knocking down Dicer1, which processes *Alu* transcripts for degradation, resulted in higher than normal *Alu* expression in retinal pigmented epithelium (RPE) cells, and this *Alu* RNA was cytotoxic, leading to RPE degeneration and disease progression (Kaneko et al., 2011). Furthermore, inhibition of the *Alu* RNAs by antisense oligos reversed this cytotoxic phenotype.

The adult *Drosophila* brain also shows retrotransposon activity in certain mushroom body neurons, and this is correlated with a relative lack of the piRNA proteins Aubergine and Argonaute 3, which normally function to suppress TE activity post-transcriptionally in the germline (Perrat et al., 2013). This study provided evidence that the piRNA pathway may also be active in somatic tissue, and its disruption was associated with increased somatic TE expression. TE activation has also been linked to aging in *Drosophila*. Li et al. (2013) showed that expression of several TEs increases with age in the fly brain, and using a GFP engineered *gypsy* element (the *gypsy-TRAP* reporter) were able to detect somatic transposition that increased with age in the brain. Finally, disrupting the RNAi protein Ago2 caused both an increase in transposition as well as a shortening of lifespan and impairment of memory with age (Li et al., 2013). It is important to note that because of the central role of Argonaute proteins in a host of small RNA pathways, Ago depletion also leads to many effects not directly related to TE silencing, including defects in microRNA processing, development, and differentiation (O’Carroll et al., 2007; Su et al., 2009; Shekar et al., 2011). These studies examining TE regulation with age, again in multiple model systems, suggest that changes in TE expression and transposition may play an important role in the cellular dysfunction observed during organismal aging.

## CONCLUSION

A number of different lines of evidence now point to chromatin structure, and heterochromatin in particular, as being an important modulator of aging (summarized in **Table 1**). A common thread throughout many theories of aging holds that the functional decline associated with aging results from an inability to properly maintain cellular structure and function with time. The chromatin context, including nucleosome structure, histone modifications, and nuclear spatial organization, has a large influence over gene expression, and evidence is accumulating that failure to maintain proper chromatin structure may be a driver of the increased damage and disruption of homeostasis associated with aging. The chromatin changes observed with age are multifaceted, and not always consistent across species and model systems, or even cell types. For instance, as mentioned above, although numerous animal model systems have shown a decrease in both overall histone as well as heterochromatin abundance with age, one of the most prominent chromatin phenotypes of the replicative senescence model is the accumulation of heterochromatin foci with age. This suggests that the role of chromatin structure in aging is likely more complex than the earlier heterochromatin loss model of aging would suggest. There are a number of potentially relevant chromatin changes that may underlie the aging process (presented in **Figure 1**). In addition to the age-related histone loss observed in yeast and mammalian replicative senescence (Feser et al., 2010; O’Sullivan et al., 2010), components of chromatin may redistribute to other locations in the genome with age, causing changes in gene expression and/or genomic stability (**Figure 1A**). An example of this mechanism is the age-related loss of heterochromatic silencing in yeast, with concomitant relocalization of the SIR silencing complex away from telomeres and mating type loci and toward rDNA (Kennedy et al., 1997). A similar mechanism operates in mammalian cells, where SIRT1 redistributes within the genome in response to DNA damage, with consequent changes in gene expression that parallel those seen in aging (Oberdoerffer et al., 2008). Chromatin domains containing characteristic histone acetylation or methylation marks may also spread, diminish, redistribute or rearrange with age, with the local chromatin and genomic context determining the phenotypic outcome of such changes (**Figure 1B**). Examples of this mechanism are prevalent at a genomic scale in worm, fly, and mammalian studies as referenced above, although the downstream effects of such rearrangements remain largely undescribed. Changes in spatial organization of various chromatin domains within the nucleus, association, or dissociation of heterochromatin with the nuclear lamina, and breakdowns in nuclear architecture and structure are also likely to be important hallmarks of aging (**Figure 1C**). The prevalence and localization of histone variants may also change with age, and this altered nucleosome composition can influence silencing and gene expression. Given the increasingly recognized role of the RNAi machinery in establishment and maintenance of heterochromatin and silencing, this pathway represents yet another potential mechanism of age-related dysfunction (**Figure 1D**). Declines in the efficiency of small RNA processing, proper genomic targeting of RNAi, and establishment and maintenance of RNAi-based transcriptional and posttranscriptional silencing all represent potential mechanisms for aging phenotypes. Finally, of particular interest is a potential role for mobilization of retrotransposons and other TEs in age-related phenotypes. TEs are widely spread throughout most eukaryotic genomes, and are known to be regulated by cellular heterochromatinization and the RNAi pathway, making this an attractive explanation for how age-related breakdown in homeostasis in these regulatory pathways may lead to deleterious mutations and genomic instability. It is likely that the phenotypes of aging are influenced by more than one of these pathways, and different mechanisms may be acting on different locations in the genome or in different cell and tissue types. Unraveling the complexity of these mechanisms and the importance of the local chromatin and genomic context will be key to understanding the downstream effects of chromatin reorganization on the aging process.

**Table 1:** Summary of major findings of recent literature linking aging and chromatin.

Organism	Summary of observations	Reference
*S. cerevisiae*	Increased H4K16ac, decreased Sir2, loss of histones with age	Dang et al. (2009)
*S. cerevisiae*	Loss of histones with age. Overexpression of histones extends lifespan	Feser et al. (2010)
Human cells	Formation during senescence of senescence-associated heterochromatin foci, with high levels of H3K9me3, H3K27me3, and HP1	Narita et al. (2003), Zhang et al. (2005), Chandra et al. (2012)
Human cells	Enrichment in H3K4me3 and H3K27me3 marks in LADs with age. Loss of H3K4me3 at downregulated genes, loss of H3K27me3 at upregulated genes	Shah et al. (2013)
Human cells	Smoothing of chromatin—open and closed regions more similar to each other during senescence. Increased expression of numerous TEs	De Cecco et al. (2013)
*C. elegans*	H3K4 methyltransferase knockdown increases lifespan. H3K4 demethylase knockdown shortens lifespan	Greer et al. (2010, 2011)
*C. elegans*	LSD1 knockdown (H3K4 and H3K9 demethylase) increases lifespan	McColl et al. (2008)
*C. elegans*	UTX-1 knockdown (H3K27 demethylase) increases lifespan. Decline in H3K27 methylation with age	Jin et al. (2011), Maures et al. (2011)
*D. melanogaster*	H3K4me3, H3K36me3 decline with age. H3K9me3, HP1 increase and relocalize with age	Wood et al., 2010
*D. melanogaster*	Decreased heterochromatin with age, HP1 overexpression extends lifespan	Larson et al. (2012)
*D. melanogaster*	H3K27 methyltransferase knockdown increases lifespan	Siebold et al. (2010)
Mouse	Numerous tissue-specific changes in DNA methylation patterns with age	Maegawa et al. (2010)
Mouse	Decreased histone acetylation, increased histone methylation with age in cochlea	Watanabe and Bloch (2013)
Mouse	Histone methylation and acetylation changes correlate with increased memory formation in neurodegeneration model	Fischer et al. (2007)
Mouse	H4K12 hypoacetylation with age in hippocampus. HDAC inhibitor improves phenotypes	Peleg et al. (2010)
Rat	Reduced H3K9 and H4K12 acetylation with age correlates with LTP defect. HDAC inhibitor improves phenotypes	Zeng et al. (2011)
Mouse	Decreased H4 acetylation in Alzheimer’s model with LTP defects. HDAC inhibitor improves phenotypes	Francis et al. (2009), Kilgore et al. (2010)
Mouse	H4K16 hypoacetylation in progeria model, HDAC inhibition improves phenotypes and extends lifespan	Krishnan et al. (2011)
Mouse cells	Gene expression correlates with nuclear lamina colocalization	Peric-Hupkes et al. (2010)
Human cells	Nuclear lamin B1 decreases during senescence	Shimi et al. (2011), Freund et al. (2012)
Human cells	Defects in lamin A processing and nuclear structure with age	Scaffidi and Misteli (2006), McClintock et al. (2007)
*C. elegans*	Loss of peripheral heterochromatin and deterioration of nuclear structure with age. Lamin knockdown shortens lfespan	Haithcock et al. (2005)
Rat	Acute stress induces H3K9me3 and TE silencing in hippocampus	Hunter et al. (2012)
*S. cerevisiae*	Ty1 mobility and genomic instability increase with age	Maxwell et al. (2011)
*C. elegans*	*Cer1* retrotransposon expression increased with age	Dennis et al. (2012)
Mouse	DNA methylation and histone modifications (Ac/Me) change at TE loci with age	Ryu et al. (2011)
Human cells	*Alu* transcripts increase during senescence, targeting by shRNA reverses senescent phenotype	Wang et al. (2011)
Human, mouse	Dicer1 loss in age-related macular degeneration leads to increased cytotoxic *Alu* transcripts, antisense targeting of *Alu* reverses phenotypes	Kaneko et al. (2011)
*D. melanogaster*	Retrotransposons are active in fly neurons	Perrat et al. (2013)
*D. melanogaster*	TE expression and somatic transposition increases with age in fly brain. Disrupting RNAi pathway causes increase in transposition and memory defects	Li et al. (2013)

In future experiments it will be increasingly important to move on from exploring associations between chromatin structure and age and toward a causal mechanism of specifically how observed chromatin changes lead to a decline in cellular homeostasis. It is likely that increased use of genetic tools, including heterochromatin and TE-based reporters, in model systems will yield mechanistic insights into the relationship between chromatin structure and aging. In addition, the decreasing cost and increasing availability of next generation sequencing-based techniques provides the opportunity to query the localization of histone modifications, longer range chromatin interactions, and the transcription of non-canonical RNAs at the whole genome level to unravel the influence of these epigenetic effects on the aging process.

## Conflict of Interest Statement

The authors declare that the research was conducted in the absence of any commercial or financial relationships that could be construed as a potential conflict of interest.
